# The adverse effects of reduced cerebral perfusion on cognition and brain structure in older adults with cardiovascular disease

**DOI:** 10.1002/brb3.171

**Published:** 2013-09-10

**Authors:** Michael L Alosco, John Gunstad, Beth A Jerskey, Xiaomeng Xu, Uraina S Clark, Jason Hassenstab, Denise M Cote, Edward G Walsh, Donald R Labbe, Richard Hoge, Ronald A Cohen, Lawrence H Sweet

**Affiliations:** 1 Department of Psychology, Kent State UniversityKent, Ohio; 2 Department of Psychiatry and Human Behavior, Alpert Medical School of Brown UniversityProvidence, Rhode Island; 3 Department of Psychology, Idaho State UniversityPocatello, Idaho; 4 Department of Neurology, Icahn School of Medicine at Mount SinaiNew York, NY; 5 Department of Neurology, Washington University at St. LouisSt. Louis, Missouri; 6 Butler HospitalProvidence, Rhode Island; 7 Department of Neuroscience, Brown UniversityProvidence, Rhode Island; 8 Department of Physiology, Université de MontréalMontréal, Quebec, Canada; 9 Center for Cognitive Aging and Memory, McKnight Brain Institute, University of FloridaGainesville, Florida; 10 Department of Psychology, University of GeorgiaAthens, Georgia

**Keywords:** Arterial spin labeling, cardiovascular disease, cerebral blood flow, cerebrovascular disease, cognitive function, magnetic resonance imaging, neuroimaging

## Abstract

**Background:**

It is well established that aging and vascular processes interact to disrupt cerebral hemodynamics in older adults. However, the independent effects of cerebral perfusion on neurocognitive function among older adults remain poorly understood. We examined the associations among cerebral perfusion, cognitive function, and brain structure in older adults with varying degrees of vascular disease using perfusion magnetic resonance imaging (MRI) arterial spin labeling (ASL).

**Materials and methods:**

52 older adults underwent neuroimaging and were administered the Mini Mental State Examination (MMSE), the Repeatable Battery for the Assessment of Neuropsychological Status (RBANS), and measures of attention/executive function. ASL and T1-weighted MRI were used to quantify total brain perfusion, total brain volume (TBV), and cortical thickness.

**Results:**

Regression analyses showed reduced total brain perfusion was associated with poorer performance on the MMSE, RBANS total index, immediate and delayed memory composites, and Trail Making Test B. Reduced frontal lobe perfusion was associated with worse executive and memory function. A similar pattern emerged between temporal lobe perfusion and immediate memory. Regression analyses revealed that decreased total brain perfusion was associated with smaller TBV and mean cortical thickness. Regional effects of reduced total cerebral perfusion were found on temporal and parietal lobe volumes and frontal and temporal cortical thickness.

**Discussion:**

Reduced cerebral perfusion is independently associated with poorer cognition, smaller TBV, and reduced cortical thickness in older adults.

**Conclusion:**

Prospective studies are needed to clarify patterns of cognitive decline and brain atrophy associated with cerebral hypoperfusion.

## Introduction

It is well established that aging and vascular processes interact to disrupt cerebral hemodynamics (Hoth 2010; de la Torre 2012). Such effects on the cerebral circulation system are unfortunate, as past work suggests reductions in cerebral blood flow (CBF) is a known contributor to cognitive impairment in older adults. For example, cerebral hypoperfusion is associated with increased risk for neurological disorders (e.g., Alzheimer's disease, vascular dementia; Pakrasi and O'Brien 2005; Austin et al. 2011) and has also been linked with more subtle deficits in nearly all domains of cognitive function in persons with cardiovascular disease (CVD; Jefferson et al. 2007a,b; Appleman et al. 2010; Moser et al. 2012).

The inverse association between CBF and cognitive function likely stems from the adverse effects of cerebral hypoperfusion on the brain (Jefferson et al. 2007a; Jerskey et al. 2009). Reduced CBF has been shown to predict decreased brain volume in persons with diabetes (van Elderen et al. 2011). Measures of systemic perfusion (e.g., cardiac indices) also significantly correlate with abnormal brain aging (e.g., smaller brain volume, white matter hyperintensities [WMH]) in patients with cardiac disease (Jefferson et al. 2007b, 2010; Jefferson 2010). Extant evidence also shows that reduced CBF is associated with structural and functional brain abnormalities in a wide range of medical and neurological populations (e.g., Alzheimer's disease, stroke patients; Austin et al. 2011; Aoi et al. 2012).

Despite these findings, the independent effects of cerebral perfusion on cognitive function and brain structure remains poorly understood. Recent studies have used positron emission tomography (PET) and found global CBF was inversely associated with cognitive test performance among vascular disease patients; however, findings from these studies are limited by small sample sizes and lack of control for confounding comorbid medical conditions that influence neurocognitive outcomes (Kitagawa et al. 2009; Brundel et al. 2012; Moser et al. 2012). Even further, limited research has used arterial spin labeling (ASL) to examine the relationship between CBF and neurocognitive outcomes in aging older adults with CVD. This is unfortunate, as rapidly growing attention has been paid to the use of ASL imaging in detecting individuals at risk for neurodegenerative disorders (e.g., Alzheimer's disease), including conversion from normal aging to dementia (Chao et al. 2010; Alexopoulos et al. 2012; Bangen et al. 2012; Wolk and Detre 2012). Past work also shows ASL imaging is sensitive to brain perfusion abnormalities in stroke survivors even before the onset of structural brain injury – though this study was limited to sample size of three participants (Brumm et al. 2010).

In light of these findings, ASL imaging may also serve as a useful biomarker for poor neurocognitive outcomes in aging older adults with CVD at risk for cognitive impairment, though no study has examined this possibility. The purpose of the current study was to examine the independent associations among cerebral perfusion using ASL imaging, structural brain indices (e.g., volume and cortical thickness), and cognitive test performance among a larger sample of older adults with varying degrees of vascular disease. In addition to the use of ASL imaging, we also sought to extend past work by capturing the independent effects of cerebral perfusion on the brain and cognition through the adjustment of medical and demographic variables that negatively impact neurocognitive outcomes in older adults.

## Material and Methods

### Participants

The sample consisted of 52 participants with complete neuropsychological, medical, and demographic data (see Table 1). This sample size was reduced from 95 due to missing data, however, those excluded were not significantly different in terms of age (*t*(92) = −0.99, *P* = 0.32), gender (χ^2^ (1, *N* = 95) = 0.55, *P* = 0.46), education (*t*(83) = −1.70, *P* = 0.09), global cognitive status (*t*(64.97) = −0.99, *P* = 0.33), cardiac function (*t*(87) = −1.12, *P* = 0.27), or in terms of comorbid medical conditions such as diabetes (χ^2^ (1, *N* = 95) = 0.85, *P* = 0.36), though there was a trend for hypertension (χ^2^ (1, *N* = 95) = 3.83, *P* = 0.05). Participants were recruited from either outpatient cardiology offices or from advertisements in local papers. The inclusion criteria were English-speaking and normal or corrected vision at the time of testing. Potential participants were excluded for significant neurological disease (e.g., history of stroke, multiple sclerosis), moderate or severe traumatic brain injury (with loss of consciousness), diagnosis of a current psychiatric illness, history of substance abuse with subsequent hospitalization, or any contraindications for magnetic resonance imaging (MRI; e.g., some metal implants). Participants were administered a comprehensive neuropsychological battery. Institutional Review Board approval was granted and written informed consent was obtained from all participants prior to testing.

**Table 1:** Demographic and medical characteristics (*N* = 52)

Demographic characteristics	
Age, mean (SD)	65.73 (8.99)
Sex (% women)	57.7
Race (% Caucasian)	94.2
Education, mean (SD)	16.06 (2.56)
WTAR, mean (SD)	110.17 (7.52)
Medical characteristics	
Cardiac index, mean (SD)	2.80 (0.58)
Heart rate, mean (SD)	64.62 (8.83)
Angina (%)	11.5
Atrial fibrillation (%)	9.6
Coronary artery disease (%)	19.2
Myocardial infarction (%)	11.5
Heart failure (%)	9.6
Hypertension (%)	42.3
Diabetes (%)	9.6
Elevated total cholesterol (%)	53.8
ACE inhibitor (%)	30.8
Antidysrhythmics (%)	7.7
Antihyperlipidemics (%)	55.8
Antihypertensive agents (%)	55.8
Neuroimaging findings	
Total brain volume, mean (SD)	447,348.79 (47,746.92) mm^3^
Total brain cortical thickness, mean (SD)	2.42 (0.10) mm
Whole brain perfusion, mean (SD)	376.45 (54.53) mL mg^−1^ sec^−1^

ACE, angiotensin-converting enzyme; WTAR, Wechsler Test of Adult Reading.

### Procedures

#### Arterial spin labeling

All scans were performed using a 3 T Siemens Tim Trio scanner (Siemens, Erlangen, Germany) located on the Brown University campus. A 32 channel head receive array was used with body resonator transmit coil, and participants were placed head first in the supine position. Foam pads were placed in the space around the head to limit motion, and participants were provided with hearing protection in the form of foam earplugs and headphones.

Following acquisition of a three-axis localizer scan, a 3D *T*_1_-MPRAGE scan was acquired with 1 mm isotropic resolution. This scan was acquired using parameters TR = 1900 msec, TE = 2.98 msec, TI = 900 msec, and readout flip angle = 9 to provide a 3D *T*_1_ image dataset for gray–white matter segmentation and morphometric analyses. ASL scans were acquired using PICORE-Q2TIPS (Wong et al. 1998; Luh et al. 1999), a Siemens product sequence that is distributed with their MRI scanners. This widely used pulsed ASL sequence has demonstrated reliability and validity (Jahng et al. 2005; Liu and Brown 2007; Noguchi et al. 2007; Petersen et al. 2010).

In brief, differences between 71 pairs of tagged and control ASL volumes were averaged to create individual mean perfusion maps. Mean perfusion values were then calculated for each individual by white matter, gray matter using *T*_1_-MPRAGE segmentation boundaries (see below). The Desikan–Killiany atlas was then used to calculate mean perfusion values for each lobe. Specifically, 71 pairs (control, perfusion weighted) of motion corrected images were averaged to provide the Δ*M* image for perfusion map computation. The first image acquired in the series served as the *M*_0_ image. An inversion slab 110 mm in thickness was placed with its proximal edge 12 mm from the inferior boundary of the imaged region. Eighteen slices of 6 mm thickness were acquired over two scans (nine slices in the first scan, eight slices in the second). In-plane voxel size was 3 mm with slice thickness of 6 mm. Timing parameters were TR = 2500 msec, TI_1_ = 700 msec, TI_2_ = 1800 msec (inversion to start of the 64^2^ echo planar image readout sequence with TE = 16 msec). Scan time for each ASL run was 4.5 min.

The *M*_0_ map for each slice was the first image acquired in the dataset. This image was not acquired with any inversion or saturation preparation and was taken with the longitudinal magnetization at full equilibrium. Δ*M* maps were formed by averaging the 71 pairs of motion-corrected images. The *M*_0_ and Δ*M* maps were used to produce perfusion maps for each slice using a Matlab script (Math Works, Natick, MA) using the expression:


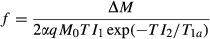


where Δ*M* is the difference signal, *M*_0_ is the equilibrium magnetization (first frame of the series), α is the inversion efficiency, TI_1_ is the interval from inversion to the double saturation pulses, TI_2_ is the interval from inversion to image readout, *T*_1a_ is the arterial blood *T*_1_, and *q* is a factor taking into account water exchange between the vascular and interstitial compartments. TI_2_ was incremented across the slices to account for the actual acquisition time for each slice relative to the inversion pulse. If individual TI_2_ values are not used for each slice, progressive underestimation of perfusion with advancing slice position in the superior direction is seen with the most distal image slice from the inversion slab showing the greatest underestimation. The tissue parameters (Stanisz et al. 2005; Zhu and Penn 2005; Wright et al. 2008) used were *T*_1a_ = 1664 msec, *T*_1T_ =1300 msec (gray matter) or 1000 msec (white matter), *T*_ex_ = 1000 msec, and λ = 0.9 mL/g. Inversion efficiency (α) was set to 0.95 based on scanner manufacturer recommendation (α = 1 corresponds to perfect inversion). The factor *q* is given by:


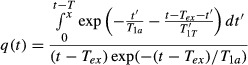



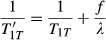


where *T*_1a_ is the arterial blood *T*_1_, *T*_1T_ is the tissue *T*_1_, *T*_ex_ is the exchange time constant, f is perfusion, λ is the blood–brain water partition coefficient, and *t* is the interval between arterial tagging and start of image acquisition. Using the above tissue parameter values results in values of *q* = 0.93 for gray matter and 0.85 for white matter. These values of *q* were applied to the perfusion calculation on a pixel basis based on gray–white matter tissue segmentation.

#### Cortical segmentation

*T*1 segmentation was accomplished by thresholding tissue probability maps to determine the boundaries of white and gray matter, which were used as region of interest masks in ASL perfusion analyses. The Desikan–Killiany atlas was also used to calculate mean ASL perfusion for each lobe. Thus, SPM5 tissue segmentation (Ashburner and Friston 2008; Ashburner 2009) was applied to the T_1_-MPRAGE data acquired during the same scanning session as the perfusion acquisitions, generating gray matter and white matter posterior probability maps for each participant in native space. The posterior probability maps were then thresholded using a minimum probability of 0.70, minimizing partial volume effects for each tissue type, yielding a binary gray matter mask and a binary white matter mask. The T_1_-weighted anatomical acquisition was processed using FreeSurfer reconstruction (Dale et al. 1999; Fischl et al. 1999), which generated separated left and right cerebral hemisphere cortical ribbon masks and cortical parcellation using the Desikan–Killiany atlas for each participant. Left and right masks were combined to form the cortical ribbon mask.

The whole brain geometry for each participant's mean perfusion data was established by concatenating the inferior 9 axial slice and superior 9 axial slice relative CBF maps generated by the scanner, along the slice (*z*) direction using Analysis of Functional Neuroimages (AFNI) (Cox 1996). The AFNI MATLAB library, freely available from http://afni.nimh.nih.gov/afni/matlab, was used to convert each whole brain perfusion array into an AFNI-compatible 3D format, having the same geometry as the whole brain relCBF dataset. The FreeSurfer cortical ribbon, anatomically based cortical parcellation (Desikan–Killiany atlas) and binary masks, were then aligned with and resampled to the same geometry as the perfusion data using AFNI/SUMA (Surface Mapping with AFNI). A whole brain perfusion map was then created using the following formula: Whole brain perfusion = (binary gray matter mask + binary white matter mask) × (perfusion data).

Alignment verification of the cortical ribbon mask, cortical parcellation, whole brain mask, and whole brain perfusion map in 3 mm × 3 mm × 6 mm space was done for each participant using the AFNI viewer. Following alignment and resampling, the mean and standard deviation (SD) of all perfusion values between 1 and 100 was calculated for each region of interest (as outlined in the Desikan–Killiany atlas), the cortical ribbon, and the whole brain. Left and right hemisphere frontal, temporal, parietal, and occipital lobe perfusion composites were also computed that consisted of the sum of the perfusion values of the respective regions of interest for each lobe.

#### Morphometric analyses

For morphometric analyses, T1 volumes were segmented into gray, white, cerebrospinal fluid, and nonbrain tissues using the FreeSurfer software package. Gray and white matter volume and thickness were then calculated and Desikan's atlas was applied to calculate thickness and volume by atlas region. These procedures are based on established techniques and procedures for the surface and subcortical reconstruction and have been described previously (Fischl and Dale 2000; Fischl et al. 2004; Han et al. 2006). The fully automated FreeSurfer v5.0 recon-all processing stream was completed for 52 participants. After preprocessing, results underwent quality control to confirm absence of any errors or defects in the segmentation. Summary composites of volume and the mean of cortical thickness of each brain region including frontal, temporal, parietal, and occipital were also calculated using the organization schema as described in Desikan et al. (2006).

#### Physiological examination

Participants' weights and heights were measures to calculate body mass index (BMI) using the standard formula: (mass in kg)/(height in meters)^2^. A transthoracic echocardiogram was conducted with two-dimensional apical views from each participant according to standards of the American Society of Echocardiography (Lang et al. 2005). Cardiac index was calculated by dividing cardiac output by BMI, which yielded a measure of cardiac output that controlled for body size.

#### Neurocognitive assessment

All participants completed a standardized neuropsychological assessment by trained research assistants under the supervision of a licensed clinical neuropsychologist. For the purposes of the current work, the primary cognitive measures included the Repeatable Battery for the Assessment of Neuropsychological Status (RBANS; Randolph et al. 1998) and the Mini Mental State Examination (MMSE; Folstein et al. 1975). The RBANS is a brief battery that consists of 10 subtests that divide into five index scores: (1) Immediate memory – leaning verbal information presented in list and story formats; (2) Language – confrontation naming and semantic fluency; (3) Visuospatial/Construction – production of a complex figure and a modified judgment of line orientation; (4) Attention – digit span and coding; (5) Delayed Memory – recall of verbal learning tasks and complex figure after a brief delay. Performance on this measure is reported in Standard Scores with a mean of 100 and standard deviation of 15. Finally, the MMSE was used to assess global cognitive function. It is a brief screening measure that assesses aspects of attention, orientation, memory, language, and calculation (Folstein et al. 1975). The range of scores extends from 0 to 30. To more fully capture attention/executive function in the sample participants were also administered the Trail Making Test A and B (TMT A and B; Reitan 1958). The TMT A requires participants to connect a series of letters in sequential order as quickly as possible. TMT B asks participants to alternately connect a series of letters and numbers as quickly as possible.

#### Estimated premorbid intelligence

To assess premorbid intelligence, the Wechsler Test of Adult Reading (WTAR) was administered to all participants. The WTAR is recognized as a valid method to estimate premorbid intelligence (Green et al. 2007). During this test, individuals are asked to read aloud a list of 50 irregularly pronounced words. Estimated IQ based on the WTAR was used in the current analyses to control for the effects of premorbid intelligence on neurocognitive function. Premorbid IQ taps into innate intelligence and is a sensitive marker of cognitive reserve (Alexander et al. 1997).

#### Demographic and medical characteristics

The patient's medical history and currently prescribed medications were self-reported during two interviews and confirmed by medical records when possible. Medications were categorized by class and those identified as cardiovascular medications were reviewed and confirmed by a clinical cardiologist.

### Statistical analyses

All RBANS composite scores were converted to standard scores (i.e., a distribution with a mean of 100 and a standard deviation of 15) adjusted for age using normative values. Normative data for the RBANS composite was used in order to facilitate clinical interpretation of cognitive status in the current sample. TMT A and B scores were also converted to standard scores for similar reasoning. A composite of gray matter volume and cortical thickness of each brain region including frontal, temporal, parietal, and occipital lobes were calculated using the organization schema as described in Desikan et al. (2006). Total brain volume (TBV) and thickness consisted of the sum and average of the frontal, temporal, parietal, and occipital lobe gray matter, respectively. All analyses examining brain volume and thickness were adjusted for intracranial volume. Lastly, a total brain perfusion composite was also computed that consisted of the mean perfusion to the left and right hemispheres of the frontal, temporal, parietal, and occipital lobe gray matter. For analyses examining regional perfusion, the average of the left and right hemispheres for each lobe was computed. One participant exhibited missing data for occipital lobe perfusion and total brain perfusion for this participant consisted of the average of the remaining lobes. There was also one case with missing data on TMT B and this case was excluded from analyses examining this cognitive test as the dependent variable.

Separate multivariable hierarchical regression analyses were performed for the MMSE, each RBANS composite, TMT A, and TMT B. For each model, demographic and medical characteristics were entered in block 1 that included: Age, sex (−1 = male; 1 = female), premorbid intelligence (as estimated by the WTAR), cardiac index, heart rate, diagnostic history of hypertension, diabetes, and atrial fibrillation (1 = positive history; 0 = negative history). Total brain cerebral perfusion was then entered into block two of each model to determine its incremental predictive validity on cognitive function. To clarify significant findings, follow-up regression analyses controlling for the above medical and demographic factors were then conducted that examined perfusion to brain lobes responsible for the mental abilities that demonstrated a significant association with total brain perfusion.

Regression analyses then examined the predictive validity of cerebral perfusion on TBV and total brain cortical thickness after accounting for the above-mentioned medical and demographic variables in addition to intracranial volume. A final series of regression analyses controlling for medical and demographic characteristics and intracranial volume were also performed to determine whether TBV and total brain cortical thickness predicted the MMSE, RBANS total index composite scores, and TMT A and B. Of note, comorbid vascular risk factors (e.g., diabetes, hypertension, atrial fibrillation, cardiac dysfunction) introduce multiple physiological processes that adversely impact cognition and brain structure in older adults. In-turn, this study included the aforementioned medical and demographic variables as covariates in order to identify the independent effects of cerebral perfusion on neurocognitive outcomes in older adults.

## Results

### Sample medical characteristics

The sample demonstrated an average cardiac index of 2.80 (SD = 0.58). Overall, 19.2% of the sample exhibited a positive diagnostic history of coronary artery disease, 11.5% angina, 11.5% myocardial infarction, and 9.6% had a heart failure diagnosis. CVD risk factors were also prevalent with nearly 42.3% of the sample having hypertension and 53.8% elevated total cholesterol. Prescribed CVD medication was also prevalent in this sample with more than half of participants prescribed antihyperlipidemics and antihypertensive agents. See Table 1 for complete medical and demographic characteristics of the sample.

Bivariate correlations examined the associations between cortical lobar cerebral perfusion and key CVD variables, including cardiac index, heart rate, and hypertension. Analyses revealed that hypertension was associated with reduced temporal lobe (*r*(50) = −0.36, *P* = 0.01) and occipital lobe perfusion (*r*(49) = −0.36, *P* = 0.01). Increased heart rate was also associated with decreased frontal lobe (*r*(50) = −0.27, *P* = 0.06) and occipital lobe perfusion (*r*(49) = −0.29, *P* = 0.04). Lastly, reduced cardiac index demonstrated a trend with lower cerebral perfusion of the temporal lobe (*r*(50) = 0.23, *P* = 0.097). No other significant findings between cerebral perfusion and the above CVD markers emerged (*P* > 0.05 for all).

### Cognitive status

The average MMSE score of the current sample was 29.06 (SD = 1.46). Similar to MMSE performance, the sample exhibited an average RBANS total index score of 106.40 (SD = 12.80). However, examination of the RBANS composites showed that many participants exhibited impairments across multiple domains of cognitive function with the most prevalent deficits found on the RBANS visuospatial/construction composite (15.4%). Impairments on TMT A and B were less common (see Table 2).

**Table 2:** Descriptive statistics of cognitive test performance (*N* = 52)

Cognitive test variable	Mean (SD)	% 1.5 SD below normative average	Range
RBANS immediate memory	105.60 (13.48)	7.7	73–129
RBANS visuospatial/construction	104.83 (15.56)	15.4	60–136
RBANS language	103.88 (11.75)	1.9	80–133
RBANS attention	105.12 (13.32)	7.7	68–138
RBANS delayed memory	103.40 (12.489)	7.7	60–126
RBANS total index	106.40 (12.80)	5.8	73–136
Attention/executive function^1^			
Trail Making Test A	11.98 (2.55)	3.8	3–16
Trail Making Test B	11.59 (2.74)	5.9	3–15

1 Scores for Trail Making Test A and B are scaled scores; one participant exhibited missing data on Trail Making Test B.

### Cerebral perfusion and cognitive function

Block 1 of the model examined the association between medical and demographic variables with the MMSE, each RBANS composites, and TMT A and B. Taken together, medical and demographic variables demonstrated a significant association with the RBANS immediate memory composite (*F*(8, 43) = 2.73, *P* = 0.02) and a trend for the MMSE (*F*(8,43) = 2.05, *P* = 0.06). Block 2 then examined the association between total brain perfusion with the MMSE, each RBANS composite, and TMT B after accounting for medical and demographic variables entered in block 1. Total brain perfusion exhibited significant associations with the following cognitive variables: MMSE, RBANS immediate memory composite, RBANS delayed memory composite, RBANS total index composite, and TMT B. In each case, reduced cerebral perfusion was associated with poorer cognitive function. No such pattern emerged for any of the other RBANS composites or TMT A (*P* > 0.05 for all). Refer to Table 3.

**Table 3:** Hierarchical multiple linear regression models examining the predictive validity of total brain perfusion on cognitive function (*N* = 52)

	Cognitive variables								
	
	Immed. mem.	Visuo	Lang	Atten.	Delay memory	Total	MMSE	TMTA	TMTB
	*β* (SE)	*β* (SE)	*β* (SE)	*β* (SE)	*β* (SE)	*β* (SE b)	*β* (SE)	*β* (SE)	*β* (SE)
Block 1									
Age	−0.05 (0.21)	0.06 (0.29)	0.01 (0.20)	−0.05 (0.22)	0.00 (0.23)	0.06 (0.22)	−0.29 (0.02)	−0.03 (0.05)	−0.21 (0.05)
Sex	0.14 (1.89)	0.02 (2.61)	0.22 (1.80)	0.17 (1.99)	0.03 (2.08)	0.16 (0.41)	0.23 (0.21)	0.16 (0.41)	0.05 (0.43)
WTAR	0.20 (0.24)	0.16 (0.33)	0.23 (0.23)	0.39 (0.25)*	0.15 (0.26)	0.32 (0.24)*	0.27 (0.03)	0.13 (0.05)	0.39 (0.05)
CI	0.26 (3.39)	0.02 (4.68)	0.02 (3.23)	−0.15 (3.57)	0.07 (3.73)	0.07 (3.48)	0.13 (0.38)	−0.08 (0.74)	−0.15 (0.76)
HR	0.18 (1.28)	0.07 (0.29)	−0.03 (0.20)	0.07 (0.22)	−0.08 (0.23)	0.06 (0.22)	0.02 (0.02)	0.19 (0.05)	0.11 (0.05)
HTN	−0.20 (3.65)	0.06 (5.05)	0.26 (3.49)	0.26 (3.85)	−0.09 (4.02)	0.06 (3.75)	0.16 (0.41)	0.21 (0.79)	0.14 (0.83)
DM	0.16 (6.14)	0.04 (8.48)	0.10 (5.86)	0.09 (6.46)	0.11 (6.75)	0.13 (6.30)	0.02 (0.70)	0.00 (1.33)	0.07 (1.39)
AFIB	−0.01 (5.91)	0.00 (8.16)	−0.11 (5.64)	0.02 (6.22)	0.10 (6.50)	−0.02 (6.07)	0.04 (0.67)	0.11 (1.28)	0.10 (1.32)
*R*^*2*^	0.34	0.05	0.21	0.25	0.07	0.22	0.28	0.12	0.21
*F*	2.73*	0.29	1.39	1.77	0.38	1.55	2.05	0.76	1.35
Block 2									
Perfusion	0.42 (0.03)**	0.05 (0.05)	0.23 (0.04)	0.25 (0.04)	0.34 (0.04)*	0.35 (0.04)*	0.31 (0.00)*	0.22 (0.01)	0.41 (0.01)*
*R*^*2*^	0.46	0.05	0.24	0.29	0.15	0.31	0.34	0.16	0.32
*F* for ΔR^2^	9.22**	0.08	1.90	2.44	3.92*	5.08*	4.11*	1.66	6.70*

Immed. memory, immediate memory; Visuo, visuospatial/construction; Lang, language; Atten, attention; Total, total index; TMT, Trail Making Test; WTAR, Wechsler test of adult reading; CI, cardiac index; HR, heart rate; HTN, hypertension; DM, diabetes mellitus; AFIB, atrial fibrillation. Sample size for TMT B = 51.

* *P* ≤ 0.05.

** *P* ≤ 0.01.

### Regional cerebral perfusion and cognitive function

In light of the specific associations between total brain perfusion with memory performance and TMT B, follow-up hierarchical regression analyses were conducted to examine the association between cerebral perfusion to cortical lobes important for learning, memory, and executive function (e.g., frontal and temporal lobe) with the RBANS immediate and delayed memory composite and TMT B. After controlling for medical and demographic variables, reduced cerebral perfusion of both the frontal (*β* = 0.51, *P* < 0.01; *R*^2^ = 0.53) and temporal lobe (*β* = 0.29, *P* = 0.05; *R*^2^ = 0.39) was associated with poorer performance on the RBANS immediate memory composite. Decrease perfusion to the frontal lobe also demonstrated an association with worse performance on the RBANS delayed memory composite (*β* = 0.32, *P* = 0.06; *R*^2^ = 0.14), though there was no association between the temporal lobe and the RBANS delayed memory composite (*β* = 0.24, *P* = 0.19; *R*^2^ = 0.10). Similarly, reduced frontal lobe perfusion exhibited significant predictive validity for poorer performance on the TMT B (*β* = 0.55, *P* = 0.02; *R*^2^ = 0.37).

### Cerebral perfusion and magnetic resonance imaging findings

After adjustment of medical characteristics, demographic variables, and intracranial volume entered in block 1, the second block of the model with total brain perfusion exhibited significant predictive validity for TBV and total brain cortical thickness. Decreased CBF was associated with smaller TBV and reduced cortical thickness. See Table 4 for a full summary of cerebral perfusion and MRI regression analyses. TBV and total brain cortical thickness were not associated with the MMSE, RBANS total index composite, or TMT A or B performances (*P* > 0.05 for all).

**Table 4:** Hierarchical multiple linear regression models examining the predictive validity of total brain perfusion on total brain volume and total brain cortical thickness (*N* = 52)

	MRI indices	
	
	Total brain volume	Total brain cortical thickness
	*β* (SE b)	*β* (SE b)
Block 1		
Age	−0.39 (404.65)**	−0.48 (0.00)**
Sex	−0.11 (4288.64)	0.00 (0.02)
WTAR	0.03 (448.37)	0.08 (0.00)
CI	0.00 (6434.95)	−0.05 (0.03)
HR	0.10 (399.93)	0.05 (0.00)
HTN	0.03 (6929.64)	−0.13 (0.03)
DM	0.13 (11,824.38)	0.08 (0.05)
AFIB	−0.01 (11,185.86)	0.18 (0.05)
Intracranial volume	0.73 (0.03)**	−0.01 (0.00)
*R*^*2*^	0.82	0.27
*F*	20.62**	1.69
Block 2		
Perfusion	0.16 (67.69)*	0.35 (0.00)*
*R*^*2*^	0.83	0.35
*F* for ΔR^2^	4.38*	5.25*

WTAR, Wechsler test of adult reading; CI, cardiac index; HR, heart rate; HTN, hypertension; DM, diabetes mellitus; AFIB, atrial fibrillation; *β,* standardized beta coefficient; *SE b,* standard error for unstandardized beta coefficient.

* *P* ≤ 0.05.

** *P* ≤ .01.

### Cerebral perfusion and cortical lobar volume and thickness

Follow-up regression analyses controlling for medical characteristics, demographic variables, and intracranial volume were performed to examine the association between cerebral perfusion and cortical lobar volumes and thickness. Significant associations were found between cerebral hypoperfusion with decreased volume for the temporal lobe (*F*(1, 41) = 12.92, *P* = 0.01; *β* = 0.25) and a strong trend for the parietal lobe (*F*(1, 41) = 3.56, *P* = 0.07; *β* = 0.19). No such pattern emerged for the frontal or occipital lobe (*P* > 0.10 for all). Decreased CBF was also associated with reduced frontal (*F*(1, 41) = 5.23, *P* = 0.03; *β* = 0.36) and temporal (*F*(1, 41) = 9.91, *P* < 0.01; *β* = 0.44) cortical thickness. There was no association between brain perfusion with parietal or occipital cortical thickness (*P* > 0.10 for all).

## Discussion

Consistent with past work, cognitive dysfunction was evident in this representative sample of older adults with CVD. The current study extends past findings by showing that CBF as assessed through ASL is associated with cognitive function and also correlated with measures of cerebral morphometry in older adults, even after controlling for key medical and demographic factors. Several aspects of these findings warrant brief discussion.

The current study suggests that reduced cerebral blood is associated with poorer cognitive function, particularly on tests of memory and attention/executive function. These findings are consistent with past work that shows the adverse impact of reduced CBF on cognitive function in vascular disease and neurological populations (e.g., Alzheimer's disease; Moren et al. 2005; Moser et al. 2012). The specific adverse effects of hypoperfusion on memory performance in the current sample is interesting in light of recent work also employing ASL imaging that suggests altered cerebral hemodynamics is a significant contributor to the pathogenesis of Alzheimer's disease (Austin et al. 2011; Alexopoulos et al. 2012; Bangen et al. 2012). This pattern is noteworthy given the elevated risk of Alzheimer's disease in persons with CVD (Qiu et al. 2006). Indeed, a specific correlation between temporal lobe perfusion and memory emerged in the current sample. The temporal lobe consists of regions of the brain that help mediate memory abilities (e.g., hippocampus) and are sensitive to the effects of aging and also particularly susceptible to hypoxic episodes stemming from fluctuations in CBF levels (Ruittenberg et al. 2005). Prospective studies are needed to fully clarify the exact role of cerebral perfusion in memory decline, including its role in the development of Alzheimer's disease.

The current study also found a specific association between cerebral perfusion of the frontal lobe and performance on a test of executive function. This is noteworthy, as executive dysfunction is common in older adults with vascular disease (Roman et al. 2004) and may be a result of reduced oxygenation to the highly plastic frontal lobes subsequent to disrupted cerebral hemodynamics. It is also possible that memory deficits in this sample may involve frontal-subcortical dysfunction (e.g., encoding, organizing) given the current association between frontal lobe perfusion and memory (Bonelli and Cummings 2008). Similarly, successful aging is commonly characterized by preserved prefrontal activation, which also corresponds to better memory on cognitive testing (Rosen et al. 2002). Nonetheless, hypoperfusion is believed to be sensitive to the early stages of cognitive impairment (Austin et al. 2011) and prospective studies are needed to elucidate patterns of cognitive decline that corresponds with cerebral hypoperfusion in aging and CVD populations.

The current findings also demonstrated an association among cerebral perfusion and smaller TBV and reduced cortical thickness. Although the cross-sectional design of the current study precludes interpretation of directionality, such findings raise the possibility that cerebral hypoperfusion is a significant contributing factor to adverse brain changes. However, future work is needed to clarify this possibility, as it is also possible that the development of vascular lesions (e.g., WMH) disrupts cerebral perfusion (Bastos-Leite et al. 2008). Brief disruptions in CBF are maintained in healthy individuals through autoregulatory mechanisms, though such mechanisms can become compromised in the presence of older age and vascular disease (Choi et al. 2006; Hoth 2010). Extant evidence suggests that such disruptions in cerebral hemodynamics may lead to adverse brain changes. For instance, cerebral hypoperfusion has been linked with accelerated brain atrophy in neurodegenerative disorders (e.g., Alzheimer's disease, Huntington's disease; Luckhaus et al. 2010; Li et al. 2010; Chen et al. 2012). Moreover, the association between reduced cerebral perfusion and cortical thickness in this study is noteworthy, as cortical thinning is a significant predictor of conversion from mild cognitive impairment to Alzheimer's disease (Querbes et al. 2009; Austin et al. 2011). The positive correlation between cerebral hypoperfusion and the temporal lobe structure in the current study also provides possible support for altered cerebral hemodynamics as a risk factor for dementia-related processes, though this awaits empirical test using longitudinal study designs. Indeed, prospective studies are needed to elucidate the potential negative impact of cerebral hypoperfusion on brain structure and associated risk with neurological changes (e.g., Alzheimer's disease).

The novelty of ASL imaging used in the current study deserves brief discussion. ASL MRI imaging has become increasingly popular method to assess cerebral perfusion because of its noninvasive nature (Austin et al. 2011). More importantly, ASL directly assesses CBF through the use of a magnetically labeled arterial blood water endogenous tracer (Aslop et al. 2010; Austin et al. 2011). The technological and also economic benefits of ASL may be advantageous over other imaging modalities that assess cerebral perfusion (e.g., PET, single-photon emission computed tomography (SPECT)), though future studies should examine ASL versus PET versus SPECT measured blood flow in older adult CVD patients as they relate to cognition and adverse brain changes.

The generalizability of the current findings is limited in several ways. First, the current study consisted of cross-sectional analyses and prospective studies are needed to determine whether cerebral hypoperfusion leads to cognitive decline and accelerated brain atrophy and cortical thinning in older adults. However, the suggested direction of these effects over time is supported by past work (Kitagawa et al. 2009). In addition, the current study found no association between brain volume or cortical thickness and cognitive function, and additional work is needed to clarify this pattern. Indeed, range restriction may have limited the current findings, as this sample exhibited relatively intact cognition and future studies with larger more diverse samples would increase the external validity. Consistent with this notion, the current study attempted to control for key medical covariates that influence neurocognitive outcomes, though larger sample sizes are needed to confirm our findings through increased statistical power and subsequent adjustment of other important possible confounds (e.g., white matter lesions, medication side effects). Similarly, prospective studies should examine the role of CBF in the development of white matter lesions, as recent work in CVD patients shows that WMH may be a key contributor to cognitive impairment (Alosco et al. 2013). Likewise, it is also possible that WMH leads to reduced CBF to exacerbate brain injury and cognitive impairment, as suggested by past work using ASL imaging in elderly subjects with diffuse confluent WMH (Bastos-Leite et al. 2008). Consistent with this notion, future work should also quantify and examine the contribution of silent infarcts and brain microbleeds to neurocognitive outcomes in aging CVD populations, particularly as they affect cerebral perfusion and subsequent neurocognitive outcomes. Lastly, cerebral perfusion may also be a more sensitive marker of early cognitive impairment relative to subclinical cerebral atrophy in the context of the normal aging process.

In brief summary, the current study found that reduced cerebral perfusion as measured by ASL is associated with poorer neurocognitive function in older adults, including reduced cognitive function, smaller TBV, and reduced cortical thickness. Although the current study supports the widely proposed etiology of cerebral perfusion in older adults, prospective studies are needed to confirm our findings and better clarify the patterns of cognitive decline and brain atrophy associated with cerebral hypoperfusion.
